# Higher vitamin B_6_ dietary consumption is associated with a lower risk of glaucoma among United States adults

**DOI:** 10.3389/fnut.2024.1363539

**Published:** 2024-06-05

**Authors:** Ziling Yang, Jinming Zhang, Yajuan Zheng

**Affiliations:** ^1^Department of Ophthalmology, The Second Hospital of Jilin University, Jilin University, Changchun, China; ^2^The First Hospital of Jilin University, Jilin University, Changchun, China

**Keywords:** cross-sectional study, glaucoma, vitamin B_6_, National Health and Nutrition Examination Survey, nutrition

## Abstract

**Objective:**

Although numerous studies have substantiated the neuroprotective effects of vitamin B_6_ on the optic nerve and its enhancement of visual function, comprehensive data delineating the correlation between vitamin B_6_ and glaucoma at a national demographic scale remain insufficient. This study is designed to explore the link between the dietary consumption of vitamin B_6_ and glaucoma.

**Methods:**

This study included 3,850 individuals aged 40 and older from the National Health and Nutrition Examination Survey (NHANES), spanning 2005–2008. Dietary consumption of vitamin B_6_ was calculated from the average of two 24-h dietary recall interviews. Glaucoma was diagnosed in accordance with the established Rotterdam criteria. To evaluate the relationship between vitamin B_6_ dietary consumption and the risk of glaucoma, we employed Restricted Cubic Splines and weighted multivariable logistic regression analysis. We employed stratified and three other sensitivity analyses to confirm the robustness of our results, and conducted a preliminary exploration of the potential association between vitamin B_6_ supplement consumption and glaucoma risk.

**Results:**

After adjusting for covariates, we found a significant inverse correlation between dietary consumption of vitamin B_6_ and glaucoma risk (*p*_non-linearity_ = 0.18; *p* for trend = 0.02). Stratified analysis and three other sensitivity analyses revealed stability in the outcomes (all p for interaction>0.05). Compared to the lowest quartile of consumption (≤1.23 mg/day), individuals in the highest quartile of vitamin B_6_ consumption (>2.34 mg/day) experienced a 75% reduction in glaucoma risk (OR = 0.25, 95% CI 0.07–0.92). However, the effect of vitamin B_6_ supplements on glaucoma was inconclusive.

**Conclusion:**

A diet high in vitamin B_6_ inversely correlates with glaucoma risk, suggesting that increasing dietary intake of vitamin B_6_ could be a viable preventative strategy against glaucoma among adults in the United States.

## 1 Introduction

Glaucoma, a leading global cause of irreversible vision loss, affects over 80 million individuals worldwide (1, 2). This condition is characterized by the progressive degeneration of retinal ganglion cells (RGCs) and their axons. Key risk factors for glaucoma include advanced age and elevated intraocular pressure (IOP) (3). The prevailing treatment strategy for glaucoma primarily revolves around the reduction of IOP. However, a significant subset of patients who effectively manage their IOP continue to experience progressive visual impairment. This phenomenon has sparked intense scientific investigation into alternative therapeutic approaches.

In glaucoma, RGC death occurs through apoptosis, triggered by various mechanisms such as mechanical damage and ischemic changes due to high IOP, both contributing to oxidative stress. Additional glaucoma risk factors, such as advanced age, genetic predispositions, and inflammatory processes, also precipitate oxidative stress through distinct biological pathways (4–7). RGCs, with their high energy demands, are particularly vulnerable to fluctuations in cellular fuel supply (8). Oxidative stress can lead to mitochondrial dysfunction and compromised ATP synthesis, causing irreparable cellular damage and ultimately resulting in the loss of RGCs (9–13). Furthermore, research suggests that oxidative stress may exacerbate damage to the trabecular meshwork (TM) in the eye, thereby increasing IOP and perpetuating a vicious cycle (14). Hence, oxidative stress is instrumental in the pathogenesis of glaucoma, constituting a crucial element of the alterations associated with glaucoma.

Emerging evidence suggests that antioxidant-rich diets, serving as an alternative therapy, can play a minimally invasive role in managing disease progression and significantly prevent glaucoma (15, 16). Specifically, vitamin B_6_, known for its antioxidant properties, shows potential in neuroprotection and improving visual function (15, 17). For instance, animal model experiments by Wang et al. (18) have revealed that vitamin B_6_ can counteract neuronal death in adult primate retinas following ischemia. Meanwhile, clinical trials by Mallone et al. (19) indicate that high-dose administration of vitamins B_1_, B_6_, and B_12_ significantly enhances visual function metrics in cases of chronic visual impairment associated with multiple sclerosis. Furthermore, Ruamviboonsuk et al. (20) have proposed that a 6-month regimen combining vitamins B_6_, B_9_, and B_12_ significantly enhances retinal sensitivity and thickness in patients with mild to moderate non-proliferative diabetic retinopathy Additionally, a case report by Xuan Cui et al. suggests that supplementation with vitamin B_6_ positively affects the delay of Gyrate atrophy (21).

Concurrently, the relationship between vitamin B_6_ and the broader group of B vitamins with glaucoma is increasingly being studied and discussed at various levels. Li et al. (22) conducted a meta-analysis to examine the association between serum levels of vitamins B_6_, B_12_, and D across various types of glaucoma. Their findings indicated no significant link between serum vitamin B_6_ levels and glaucoma. Similarly, meta-analyses by Xu et al. (23) and Li et al. (24) on non-ocular risk factors for primary glaucoma corroborated these results. In contrast, clinical research led by Rolle et al. (25) demonstrated that supplements containing vitamins B_2_, B_6_, and folic acid could decelerate the progression of functional impairment in patients with primary open-angle glaucoma and enhance visual function. Additionally, a recent cross-sectional study by Lee et al. (26) exploring the relationship between niacin intake and glaucoma incorporated the intake of vitamins B_2_ and B_6_ as covariates in their model. However, direct research linking dietary intake of vitamin B_6_ to glaucoma is relatively scarce and tends to focus on specific types of the condition (27), such as the prospective study by Walter Willet and others on exfoliative glaucoma and its suspected cases in relation to folic acid, vitamin B_6_, and B_12_ (27). As a nationally representative cross-sectional study, this research aims to utilize NHANES data to explore the potential relationship between dietary vitamin B_6_ intake and glaucoma in the U.S. population.

## 2 Materials and methods

### 2.1 Study population

**Figure 1:**
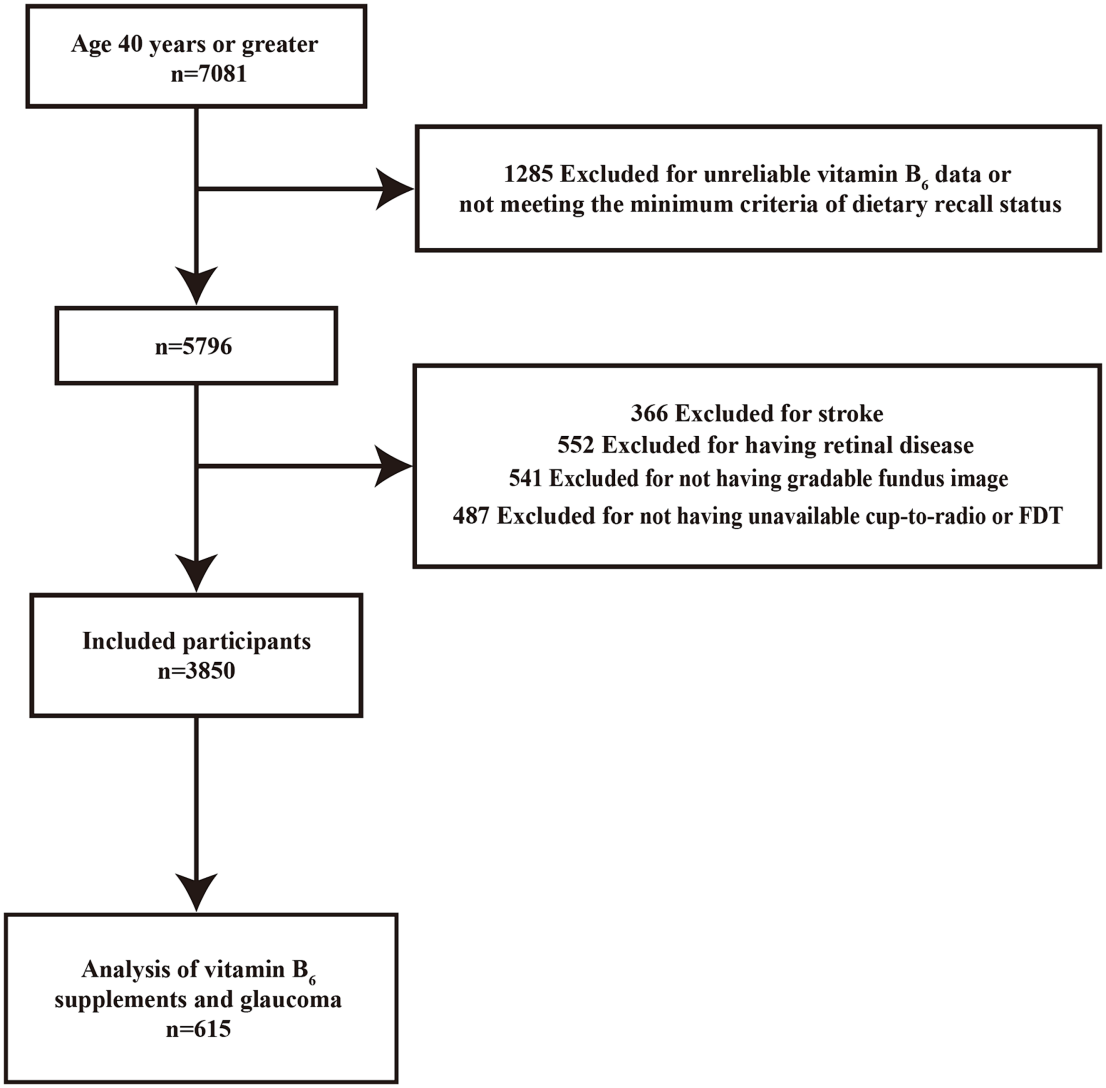
Flowchart depicting the selection process for the final inclusion group.

The National Health and Nutrition Examination Survey (NHANES), conducted biennially, is a national epidemiological cross-sectional study that utilizes a stratified multistage cluster sampling design to collect health and nutrition data from the U.S. population (28). Additional information about this survey is available on its official website (29). This study adheres to strict ethical standards, ensuring that all participants voluntarily provided informed consent. Our research utilized data collected from 2005 to 2008 by NHANES, involving 7,081 individuals aged 40 and older who underwent eye examinations. We excluded participants who did not attend two dietary interviews or lacked vitamin B_6_ dietary data (*n* = 1,285), those without gradable fundus photographs (*n* = 541), participants with unavailable or unusable frequency doubling technology (FDT) visual field test results or cup-to-disc ratio data (*n* = 487), and those with abnormal FDT results or cup-to-disc ratios likely due to alternative causes such as cerebrovascular disease (*n* = 366) or various retinal diseases (*n* = 552). After these exclusions, 3,850 participants were included in the final analysis. Given that the missing values in all confounding variables were less than 5% of the final sample size, based on existing literature (30), we considered such a small proportion of missing data unlikely to introduce significant bias into our results. Therefore, we did not exclude these participants from our analysis. Baseline characteristics of included and excluded participants are detailed in Supplementary Table S1. Of the final sample, data on vitamin B_6_ supplement intake was available for 615 participants; this data was analyzed to investigate the association between vitamin B_6_ supplement intake and the incidence of glaucoma (Figure 1).

### 2.2 Exposure and outcome variables

The principal exposure in this study is the intake of vitamin B_6_ in daily diets, derived from the Dietary Interview component of the dataset. This dataset focuses on collecting and analyzing dietary habits and nutrient intake across the U.S. population (31, 32). In our study, all eligible participants underwent two 24-h dietary recall interviews. These interviews detailed the types and quantities of food consumed in the 24 h preceding each interview. The initial interview was conducted at the Mobile Examination Center (MEC) (32), followed by a second interview via telephone 3 to 10 days later (33). We calculated the average of two interview sessions to estimate the final intake of vitamin B_6_, which potentially provides a closer approximation of the participants’ usual dietary intake compared to using data from a single interview.

The main result was the identification of glaucoma according to the Rotterdam Criteria, which considered the participants’ abnormalities in the visual field and the appearance of the optic nerve. In NHANES, optic nerve morphology was evaluated by optic nerve imaging, and FDT was used to measure glaucoma visual field defects. Glaucoma was clinically diagnosed if at least one eye exhibited a positive FDT result, combined with a CDR in one eye or CDR asymmetry across both eyes, meeting or exceeding 97.5% of the average NHANES population’s threshold (34–36).

In NHANES, trained technicians performed the FDT examination under dark conditions. Each eye underwent testing at 19 visual field locations, ensuring comprehensive assessment. The FDT outcome was classified as positive (using the 2–2-1 algorithm) if a minimum of two locations fell beneath the 1% threshold level in both initial and subsequent tests, with at least one identical failed location in both assessments (37). The CDR was ascertained through the analysis of two 45° non-mydriatic retinal digital images, captured by proficient technicians. Each image underwent review by a minimum of three trained graders. If the CDR scores from at least two of the three graders deviated by no more than 0.1, the outcome was established based on the median score. If the score difference between any two graders is greater than 2.2, the image was reviewed again with all graders present to reach a consensus (38).

### 2.3 Covariates

We conducted an extensive review of existing clinical research and practices related to glaucoma, including 20 potential confounding variables in our model to ensure reliability. These variables include age, sex, race, marital status, education level, household income, total caloric intake, Body Mass Index (BMI), alcohol consumption, waist circumference, diabetes, hypertension, cardiovascular disease (CVD), serum total cholesterol, C-reactive protein (CRP) levels, and dietary consumption of vitamins B_1_, B_2_, B_3_, B_9_, and B_12_ (26, 27, 35, 39, 40). The first six of these variables, pertaining to sociodemographic information, were gathered through personal interviews (41). The intake levels of these vitamins were obtained from dietary interviews, which also accounted for total energy and alcohol consumption (32, 33). For our study, CVD was defined to include any heart-related conditions, such as congestive heart failure, coronary artery disease, angina, or myocardial infarction. Diabetes was classified based on a physician’s diagnosis, the use of insulin or oral hypoglycemic agents, fasting plasma glucose levels ≥7.0 mmol/L, or glycated hemoglobin values ≥6.5%. Additionally, hypertension was characterized by a systolic blood pressure ≥ 130 mmHg or a diastolic blood pressure ≥ 80 mmHg, ascertained by the mean of three consecutive readings, a self-disclosed history of hypertension, or the administration of antihypertensive medication (42, 43). Waist circumference measurements and BMI were obtained in the MEC (44), and non-fasting blood samples collected there were sent to laboratory for analysis to obtain total serum cholesterol levels and CRP (45–47). Due to the missing values of the covariate “smoking” reaching 47% of the study population, it could introduce significant bias to the statistical results. Therefore, this variable was not included in the adjusted covariates in this study.

### 2.4 Exploratory analysis of supplemental consumption of vitamin B_6_

Vitamin B_6_ supplements were introduced as exploratory variables to evaluate the potential influence of different sources of vitamin B_6_ on the risk of glaucoma. The consumption of supplemental vitamin B_6_ was determined by averaging data from two 24-h dietary recall interviews with participants.

### 2.5 Statistical analysis

We conducted weighted analyses using dietary sample weights from NHANES, adapting these to its complex survey sampling design and multilevel clustering. Continuous variables were described using weighted means with accompanying standard errors (SE), and categorical variables were presented as frequencies. The aim was to compare the distribution of potential confounding factors between participants with and without glaucoma. To assess the impact of the distribution of variables, the continuous exposure variables of dietary and supplemental intake of vitamin B_6_ were converted into quartiles. This process involves arranging the data in ascending order based on the values of the exposure variables, and then determining the quartiles’ cutoff values at the 25th, 50th, and 75th percentiles. These cutoff values divide the dataset into four approximately equal groups, allowing evaluation of the impact of varying levels of exposure variables on the outcome. Similarly, the division into quintiles follows a similar method, with cutoff values at the 20th, 40th, 60th, and 80th percentiles.

Three analytical models were constructed: a base model without adjustment, Model 1 with adjustments for age and sex, and Model 2, which extended these adjustments to include all relevant confounders. Building on Model 2, we first analyzed vitamin B_6_ dietary consumption as a continuous variable using Restricted Cubic Splines (RCS) method. By positioning three knots at the 10th, 50th, and 90th percentiles, we used the likelihood ratio test to assess its association with glaucoma risk. Subsequently, we utilized the quartiles of dietary intake and supplemental intake of vitamin B_6_, exploring their association with the risk of glaucoma using a weighted logistic regression model.

To bolster the validity of our research outcomes, we used subgroup analysis, accounting for various dimensions including age, race, sex, marital status, household income, education level, diabetes, CVD, and hypertension. This was to assess the consistency and stability of the results across different population groups. Additionally, we conducted three types of sensitivity analyses: (1) an unweighted logistic regression analysis of the sample; (2) an analysis of vitamin B_6_ dietary intake divided into quintiles; and (3) an analysis excluding all participants with missing values in the confounding variables.

All statistical tests were conducted as two-tailed, with a significance level set at *p* < 0.05. Analytical procedures were executed using R 4.3.1.

## 3 Results

### 3.1 Baseline characteristics

The research included 3,850 unweighted participants and 96,323,492 weighted participants, all aged 40 or above, who participated in dietary interviews and had accessible fundus imaging and FDT test data. Out of these, 151 were identified as having glaucoma as per the study’s definition, making up 3.9% of the unweighted population. In the weighted population, the proportion of glaucoma was 2.6% (*n* = 2,463,459). The findings indicated that among those with glaucoma, there was a significantly higher proportion of participants who were older, non-Hispanic black, not married, and had diabetes, hypertension, CVD, lower CRP, larger waist circumference, and lower total serum cholesterol levels, along with lower intake of vitamins B_3_ and B_12_ (all *p* < 0.05). No notable differences were detected among participants in sex, household income, education level, total energy consumption, BMI, alcohol consumption, vitamin B_1_, B_2_ and B_9_ intake, irrespective of their glaucoma status (all *p* > 0.05) (Supplementary Table S2).

### 3.2 Vitamin B_6_ dietary consumption and glaucoma

**Figure 2:**
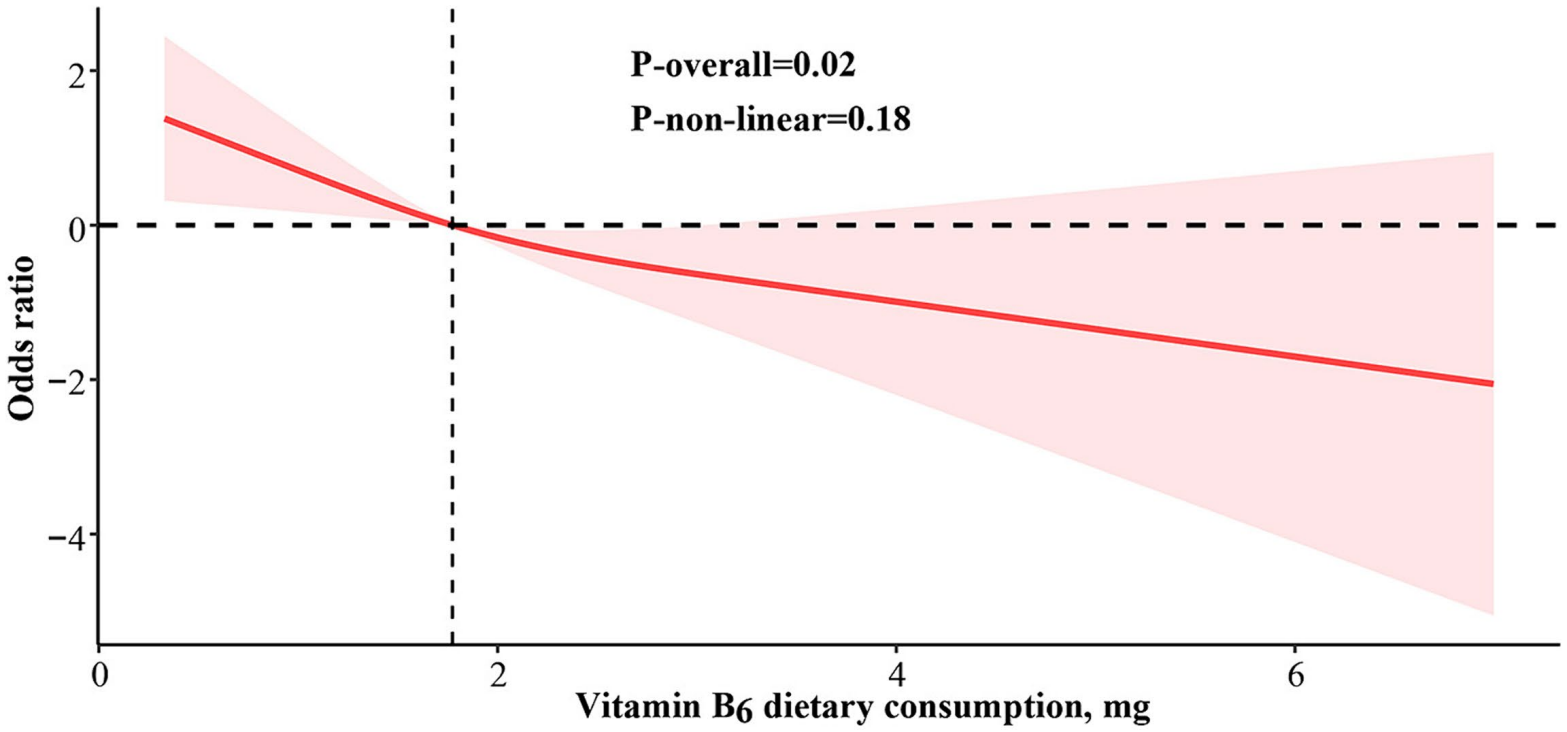
Restricted cubic spline analysis of vitamin B_6_ dietary consumption and odds ratio of glaucoma based on Model 2, which was adjusted for age, sex, race, marital status, educational level, household income, total energy consumption, BMI (body mass index), alcohol consumption, waist circumference, diabetes, hypertension, cardiovascular disease, serum total cholesterol, C-reactive protein, dietary consumption of vitamin B_1_, B_2_, B_3_, B_9_ and B_12_.

The average dietary consumption of vitamin B_6_ among all eligible participants was 1.96 mg/day (SE 0.02). Notably, the average vitamin B_6_ dietary consumption in the glaucoma group [1.82 (SE 0.10) mg/day] was markedly inferior to that in the non-glaucoma group [2.04 (SE 0.03) mg/day] (*p* = 0.02). Based on the fully adjusted Model 2, the RCS analysis indicated no significant non-linear correlation between dietary vitamin B_6_ consumption and glaucoma risk (*p*_overall_ = 0.02, *p*_non-linearity_ = 0.18) (Figure 2).

To delve deeper into the potential linear correlation between the consumption of dietary vitamin B_6_ and glaucoma, we divided vitamin B_6_ dietary consumption into four quartiles: Q1 (first) (≤1.23 mg/day), Q2 (second) (>1.23 to ≤1.70 mg/day), Q3 (third) (>1.70 to ≤2.34 mg/day), and Q4 (fourth) (>2.34 mg/day). The outcomes from the logistic regression model revealed a substantial negative linear correlation between glaucoma and vitamin B_6_ dietary consumption in the crude model (*p* for trend = 0.003), with a notable reduction in glaucoma proportion in the higher quartiles of vitamin B_6_ dietary consumption compared to the lowest quartile (Q3: OR 0.53, 95% CI 0.32–0.88; Q4: OR 0.42, 95% CI 0.24–0.75). This inverse linear correlation persisted even after controlling for covariates. (Model 1: Q3: OR = 0.50, 95% CI 0.29–0.84; Q4: OR = 0.38, 95% CI 0.20–0.73; p for trend = 0.004; Model 2: Q3: OR = 0.43, 95% CI 0.21–0.86; Q4: OR = 0.25, 95% CI 0.07–0.92 *p* for trend = 0.02). (Table 1).

**Table 1:** Results of weighted logistic regressions between vitamin B_6_ dietary consumption and risk of glaucoma.

**Vitamin B**_**6**_ **dietary Consumption, mg/day**	**Glaucoma**					
	Crude model		Model 1		Model 2	
	OR (95% CI)	*P*	OR (95% CI)	*P*	OR (95% CI)	*P*
Q1 (≤1.23)	Ref		Ref		Ref	
Q2 (1.23–1.70)	0.59 (0.34, 1.02)	0.06	0.57 (0.31, 1.02)	0.06	0.50 (0.23, 1.11)	0.08
Q3 (1.70–2.34)	0.53 (0.32, 0.88)	0.02	0.50 (0.29, 0.84)	0.01	0.43 (0.21, 0.86)	0.02
Q4 (>2.34)	0.42 (0.24, 0.75)	0.005	0.38 (0.20, 0.73)	0.01	0.25 (0.07, 0.92)	0.04
*P* for trend	0.003		0.004	0.02		

Model 1 adjusted for age and sex. Model 2 further adjusted for race, marital status, educational level, household income, total energy consumption, BMI (body mass index), alcohol consumption, waist circumference, diabetes, hypertension, cardiovascular disease, serum total cholesterol, C-reactive protein, dietary consumption of vitamin B_1_, B_2_, B_3_, B_9_, and B_12_. OR (95% CI), odds ratio (95% confidence intervals).

### 3.3 Sensitivity analysis

**Figure 3:**
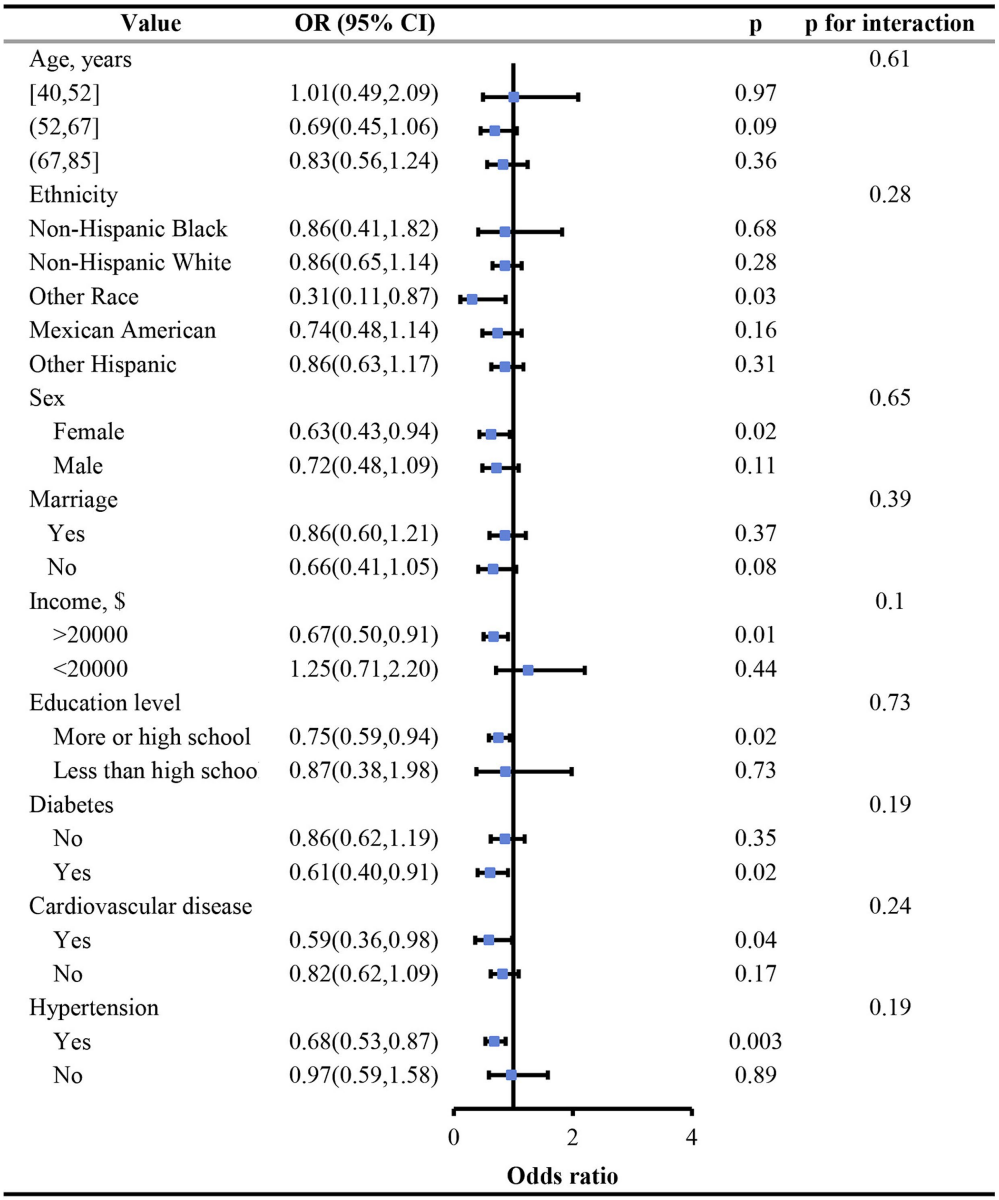
Subgroup analysis of correlation between vitamin B_6_ dietary consumption and glaucoma stratified by age, race, sex, marital status, household income, education level, diabetes, cardiovascular disease and hypertension.

Subgroup analysis revealed no significant interactions between dietary vitamin B_6_ consumption and variables such as age, race, sex, marital status, family income, educational level, diabetes, CVD, and hypertension (all *p* for interaction >0.05) (Figure 3). Additionally, in our three sensitivity analyses, the results remained robust: (1) unweighted logistic regression analysis was conducted on the sample; (2) dietary intake of vitamin B_6_ was analyzed by dividing it into quintiles; (3) analysis was performed after excluding all participants who had missing values in the confounding variables (Supplementary Tables S3–S6).

### 3.4 Vitamin B_6_ supplements shows no negative correlation with glaucoma

To investigate the impact of vitamin B_6_ supplement consumption on glaucoma, we conducted a multivariate regression analysis examining the association between vitamin B_6_ supplement intake and glaucoma incidence. The results indicated that the vitamin B_6_ supplements consumption exhibited no association with glaucoma (Table 2).

**Table 2:** Associations of vitamin B_6_ supplements and risk of glaucoma.

**Vitamin B**_**6**_ **supplements, mg/day**	**Glaucoma**					
	Crude model		Model 1		Model 2	
	OR (95% CI)	*P*	OR (95% CI)	*P*	OR (95% CI)	*P*
Q1 (≤2)	Ref		Ref		Ref	
Q2 (2, 3)	0.95 (0.24, 3.78)	0.94	0.82 (0.21, 3.18)	0.75	1.03 (0.19, 5.52)	0.97
Q3 (3–6)	0.84 (0.08, 8.95)	0.88	1.00 (0.10, 10.40)	1.00	0.36 (0.04, 3.13)	0.33
Q4(>6)	1.23 (0.45, 3.36)	0.66	1.36 (0.48, 3.83)	0.53	0.87 (0.17, 4.57)	0.87
*P* for trend	0.60		0.43		0.60	

Model 1 adjusted for age and sex. Model 2 further adjusted for race, marital status, educational level, household income, total energy consumption, BMI (body mass index), alcohol consumption, waist circumference, diabetes, hypertension, cardiovascular disease, serum total cholesterol, C-reactive protein, dietary consumption of vitamin B_1_, B_2_, B_3_, B_9_, and B_12_. OR (95% CI), odds ratio (95% confidence intervals).

## 4 Discussion

In our study involving 3,850 Americans aged 40 and older, we discovered a significant independent linear negative correlation between dietary intake of vitamin B_6_ and the risk of glaucoma. This relationship remained robust even after adjusting for multiple potential confounders. Relative to the first quartile of vitamin B_6_ dietary consumption, which falls below the Recommended Dietary Allowance (RDA) of 1.6 mg/day for men and 1.4 mg/day for women, the odds of developing glaucoma dropped by 75% for individuals in the fourth quartile (>2.34 mg/day) (OR = 0.25, 95% CI 0.07–0.92). Additionally, we found no significant correlation between consumption of vitamin B_6_ supplements and glaucoma risk.

In light of these results, our review of existing research indicates that there is still debate over whether vitamins from natural dietary sources and synthetic supplements are equivalent in terms of bioavailability and metabolic effects (48). While studies by Nelson et al. (49) suggest that vitamin B_6_ from supplements has a higher absorption efficiency, research by Meinrad Lindschinger shows that natural and synthetic B vitamins are roughly equivalent in bioavailability. However, natural vitamin B_6_ not only improves serum vitamin B_6_ concentrations more effectively compared to its synthetic counterpart but also has a stronger impact on metabolic parameters like homocysteine levels and total antioxidant capacity (48). Additionally, there is currently no evidence to suggest that consuming large amounts of vitamin B_6_ from food leads to adverse effects (50). However, multiple reports indicate that long-term excessive use of vitamin B_6_ supplements can cause adverse reactions (50–53). Additionally, the discrepancy in our findings might be attributed to significant data loss among supplement users, reducing the sample size from 3,850 to 615, potentially introducing substantial bias into the results. Given the limitations of NHANES data, we could not further explore the relationship between these variables. Considering the controversies in existing research, we believe that comparing the efficacy of different sources of vitamin B_6_ is a crucial direction for future studies.

Contrarily, our study findings diverge from those reported by JaeH Kang and his colleagues, who conducted a thorough cohort study identifying no significant link between vitamin B_6_ intake and the risk of exfoliative glaucoma or its suspected cases. We hold their work in high regard for its rigor and evidential strength (27). The variations in our results likely stem from several key factors: Firstly, our research included all types of glaucoma, not just exfoliative glaucoma, which is estimated to constitute about 25% of all open-angle glaucoma cases (54). Secondly, our method for assessing vitamin B_6_ intake, based on 24-h dietary recall interviews, differed from Kang’s team, who used a long-term average intake method. Additionally, in the study populations, Kang and colleagues included female registered nurses and male health professionals, whereas our study population is based on NHANES data and broadly involves non-institutionalized civilians across the United States. There may be some degree of demographic and sociological differences between the two study populations. These factors highlight the diversity of research designs, which may explain differences in study outcomes. These key elements are worthy of further exploration and discussion in future research.

Given the discrepancies in existing research findings, a deep understanding of the biochemical roles and mechanisms of vitamin B_6_ is particularly crucial. Vitamin B_6_, a coenzyme essential for countless biochemical reactions, comprises six compounds (55). These include pyridoxal, pyridoxamine, pyridoxine, and their 5′-phosphate esters (56). Pyridoxal-5′-phosphate (PLP), known as the biologically active form of vitamin B_6_ in humans, is often used interchangeably with the term “Vitamin B_6_” (57, 58).

One potential protective mechanism of vitamin B_6_ against glaucoma may be its antioxidant activity. Pioneering work by Margaret Daub’s team has shown that vitamins are highly effective in quenching reactive oxygen species (ROS), with potential comparable to carotenoids and tocopherols (58, 59). Vitamin B_6_, by quenching excess ROS, can reduce damage to ocular structures such as RGCs in the eye mitochondria caused by ROS imbalance, and play a certain role in the prevention of glaucoma and intervention in disease progression (13). On the other hand, vitamin B_6_ has also been found to be involved in maintaining normal homocysteine (Hcy) levels, and its circulating levels often decrease concurrently with hyperhomocysteinemia (60). High levels of Hcy can promote the progression of glaucoma by stimulating cytochrome c release and ROS production, and by inducing mitochondrial dysfunction and oxidative stress through the ERK1/2 signaling pathway (60). Emerging research increasingly substantiates that hyperhomocysteinemia is a substantial risk factor in the progression of glaucoma (60, 61). A study carried out in Russia involving participants with glaucoma and early-stage cataracts has demonstrated that a 20-day course of low-dose pyridoxine hydrochloride eye drops can impact visual parameters, such as changes in visual acuity and expansion of the visual field. This regimen also showed a reduction in intraocular pressure and the Becker’s coefficient (62).

Another potential protective mechanism of vitamin B_6_ against glaucoma may be its role in promoting the synthesis of myelin phospholipids, which have been shown to play a significant role in nourishing axons (63, 64). PLP, as a coenzyme in myelin synthesis, plays a positive role in slowing disease progression (63). Considering these potential mechanisms, it’s plausible that boosting vitamin B_6_ consumption could strengthen the resilience of RGCs against glaucomatous neurodegeneration.

This study’s strength lies in its use of stringent inclusion and exclusion criteria to enroll a large, nationally representative sample of American adults, which enhances the reliability of the results to a certain extent. Additionally, we validated the robustness of our conclusions through various sensitivity analyses, including subgroup analyses. On another note, our analysis of the relationship between vitamin B_6_ supplements and glaucoma offers a preliminary exploration of the equivalence in efficacy between different sources of vitamin B_6_.

This study presents several limitations that warrant consideration. First, to mitigate the impact of confounding factors, we implemented stricter inclusion and exclusion criteria that excluded individuals with stroke and retinal diseases. This approach limited the generalizability of our findings to the excluded groups and potentially introduced selection bias. Moreover, due to the cross-sectional nature of NHANES data, we could not explore causal or temporal associations between dietary vitamin B_6_ consumption and glaucoma risk. Dietary intake data, based on self-reporting, may be subject to recall bias. Additionally, given the potential variability in daily food consumption, using data from only two interviews may not accurately reflect participants’ regular dietary habits. Furthermore, we defined glaucoma using the Rotterdam criteria, which, despite being internationally validated, have inherent false positive and negative rates (34). The lack of comprehensive eye examinations to differentiate among the various subtypes of glaucoma also limited our understanding of the correlation between vitamin B_6_ dietary consumption and various subtypes of the condition.

To better understand the role of vitamin B_6_ in preventing and treating glaucoma, future research should employ less restrictive criteria to include a more diverse group of participants, thereby enhancing the generalizability of the findings. Additionally, more frequent dietary assessments or extended dietary tracking periods could enhance the accuracy of dietary intake estimates. It is also important to take into account other influencing factors such as genetics and environmental conditions, and to conduct further investigations through randomized controlled trials or epidemiological cohort studies. Furthermore, distinguishing between different types and stages of glaucoma will help identify the most effective ways to use vitamin B_6_ in prevention and treatment, offering more precise guidance for clinical practice.

## 5 Conclusion

Our findings indicate an inverse linear correlation between vitamin B_6_ dietary consumption and glaucoma risk. Thus, sufficient dietary consumption of vitamin B_6_ may serve as a preventive measure against glaucoma.

## Data availability statement

The datasets presented in this study can be found in online repositories. The names of the repository/repositories and accession number(s) can be found a: https://www.jianguoyun.com/p/DagglMEQ_cmbDBjDrqgFIAA.

## Ethics statement

The studies involving humans were approved by the NCHS Research Ethics Review Board. The studies were conducted in accordance with the local legislation and institutional requirements. The participants provided their written informed consent to participate in this study. The manuscript presents research on animals that do not require ethical approval for their study.

## Author contributions

ZY: Formal analysis, Methodology, Writing – original draft. JZ: Conceptualization, Writing – original draft. YZ: Conceptualization, Funding acquisition, Methodology, Writing – review & editing.
