# Supplementary material for: Higher vitamin B6 dietary consumption is associated with a lower risk of glaucoma among United States adults
Source: Front Nutr. 2024 Jun 5;11:1363539. doi: 10.3389/fnut.2024.1363539 (PMC11188593; doi:10.3389/fnut.2024.1363539)
Supplement: Supplementary file 1 [file Table_1.pdf]

Supplementary Tables 1. The comparison between the included and excluded participants. BMI, body mass index; SE, standard error.

Characteristic	Overall n=7081	Included n=3850	Excluded n=3231
Age, years, mean (SE)	60.62(0.15)	58.60(0.20)	63.04(0.24)
Sex, n (%)			
Female	3575(50.49)	1977(51.35)	1598(49.46)
Male	3506(49.51)	1873(48.65)	1633(50.54)
Race, n (%)			
Non-Hispanic white	3688(52.08)	2197(57.06)	1491(46.15)
Non-Hispanic black	1514(21.38)	698(18.13)	816(25.26)
Mexican	1103(15.58)	577(14.99)	526(16.28)
Other Hispanic	518(7.32)	264(6.86)	254(7.86)
Other races	258(3.64)	114(2.96)	144(4.46)
Marriage, n (%)			
Yes	4338(61.26)	2553(66.31)	1785(55.25)
No	2734(38.61)	1295(33.64)	1439(44.54)
Household income, $, n (%)			
<20,000	1751(24.73)	764(19.84)	987(30.55)
≥20,000	5043(71.22)	2970(77.14)	2073(64.16)
Educational level, n (%)			
Less than high school	2257(31.87)	984(25.56)	1273(39.40)
High school graduation or more	4811(67.94)	2865(74.42)	1946(60.23)
Vitamin B_1 intake, mg/day, mean (SE)	1.53(0.01)	1.58(0.01)	1.44(0.01)
Vitamin B_2 intake, mg/day, mean (SE)	2.09(0.01)	2.15(0.02)	1.95(0.02)
Vitamin B_3 intake, mg/day, mean (SE)	22.86(0.13)	23.79(0.19)	21.02(0.18)
Vitamin B_6 intake, mg/day, mean (SE)	1.90(0.01)	1.96(0.02)	1.76(0.02)
Vitamin B_9 intake, mcg/day, mean (SE)	381.37(0.13)	393.64(0.18)	357.18(0.18)
Vitamin B_{12} intake, mcg/day, mean (SE)	5.31(0.07)	5.50(0.10)	4.94(0.10)
Total energy consumption, kcal/day, mean (SE)	1909.44(9.14)	1980.86(12.72)	1768(12.43)
Waist, cm, mean (SE)	100.71(0.18)	100.55(0.24)	100.95(0.27)
BMI, kg/m^2, mean (SE)	29.16(0.08)	29.16(0.10)	29.16(0.12)
Alcohol consumption, gm/day, mean (SE)	7.00(0.21)	7.78(0.30)	5.47(0.28)
Diabetes, n (%)			
Yes	2387(33.71)	1125(29.22)	1262(39.06)
No	4405(62.21)	2720(70.65)	1685(52.15)
Cardiovascular disease, n (%)			
Yes	913(12.89)	377(9.79)	536(16.59)
No	5846(82.56)	3459(89.84)	2387(73.88)
Hypertension, n (%)			
Yes	3778(53.35)	1961(50.95)	1817(56.24)
No	3017(42.60)	1889(49.06)	1128(34.91)

C-reactive protein, mg/dl, mean (SE)	0.49(0.01)	0.46(0.01)	0.53(0.02)
Serum total cholesterol, mmol/l, mean (SE)	5.20(0.01)	5.26(0.02)	5.12(0.02)

Supplementary Tables 2. Examination of the demographic and characteristic disparities among participants with and without glaucoma. BMI, body mass index; SE, standard error.

**Characteristic**	**Non-Glaucoma (n=3699)**	**Glaucoma (n=151)**	**p Value**
Age, years, mean (SE)	55.68(0.41)	65.32(1.05)	< 0.0001
Sex, n (%)			0.14
Female	1908(55.33)	69(45.59)	
Male	1791(44.67)	82(54.41)	
Race, n (%)			< 0.001
Non-Hispanic white	2134(80.08)	63(62.35)	
Non-Hispanic black	649(8.17)	49(22.00)	
Mexican	552(4.96)	25(5.34)	
Other Hispanic	258(2.75)	6(1.12)	
Other races	106(4.04)	8(9.19)	
Marriage, n (%)			0.01
Yes	2464(70.35)	89(60.43)	
No	1233(29.65)	62(39.57)	
Household income, $, n (%)			0.23
<20,000	731(13.65)	33(17.62)	
≥20,000	2857(86.35)	113(82.38)	
Educational level, n (%)			0.37
Less than high school	944(14.92)	40(18.62)	
High school graduation or more	2754(85.08)	111(81.38)	
Vitamin B_1 intake, mg/day, mean (SE)	1.65(0.03)	1.59(0.08)	0.45
Vitamin B_2 intake, mg/day, mean (SE)	2.27(0.03)	2.14(0.12)	0.26
Vitamin B_3 intake, mg/day, mean (SE)	24.88(0.30)	22.66(1.10)	0.04
Vitamin B_6 intake, mg/day, mean (SE)	2.04(0.03)	1.82(0.10)	0.02
Vitamin B_9 intake, mcg/day, mean (SE)	409.77(7.03)	388.68(23.40)	0.36
Vitamin B_{12} intake, mcg/day, mean (SE)	5.77(0.16)	4.80(0.31)	0.002
Total energy consumption, kcal/day, mean (SE)	2054.41(21.72)	1981.73(109.79)	0.5
Waist, cm, mean (SE)	99.97(0.53)	103.34(1.14)	0.02
BMI, kg/m^2, mean (SE)	29.07(0.23)	29.59(0.53)	0.39
Alcohol consumption, gm/day, mean (SE)	8.57(0.54)	6.57(1.95)	0.36
Diabetes, n (%)			0.01
Yes	1070(23.47)	55(36.83)	
No	2624(76.53)	96(63.17)	
Cardiovascular disease, n (%)			< 0.0001
Yes	351(7.10)	26(18.66)	
No	3335(92.90)	124(81.34)	

Hypertension, n (%)			< 0.0001
Yes	1857(46.65)	104(65.96)	
No	1842(53.35)	47(34.04)	
C-reactive protein, mg/dl, mean (SE)	0.45(0.02)	0.36(0.03)	0.04
Serum total cholesterol, mmol/l, mean (SE)	5.34(0.03)	4.94(0.10)	< 0.001

Supplementary Table 3. Results of unweighted logistic regressions between vitamin B_6 dietary consumption and risk of glaucoma. Model 1 adjusted for age and sex. Model 2 further adjusted for ethnicity, marital status, educational level, household income, total energy consumption, BMI (body mass index), alcohol consumption, waist circumference, diabetes, hypertension, cardiovascular disease, serum total cholesterol, C-reactive protein, dietary consumption of vitamin B_1, B_2, B_3, B_9 and B_{12}. OR (95% CI), odds ratio (95% confidence intervals).

**Vitamin B_6 dietary Consumption, mg/day**	**Glaucoma**					
	Crude model		Model 1		Model 2	
	OR (95% CI)	P	OR (95% CI)	P	OR (95% CI)	P
Q1(≤1.23)	ref		ref		ref	
Q2(1.23-1.70)	0.73(0.47,1.13)	0.16	0.71(0.46,1.11)	0.14	0.61(0.38,1.03)	0.07
Q3(1.70-2.34)	0.69(0.44,1.07)	0.09	0.66(0.42,1.03)	0.07	0.57(0.35,1.05)	0.08
Q4(>2.34)	0.54(0.34,0.85)	0.01	0.51(0.32,0.83)	0.01	0.41(0.19,0.89)	0.02
P for trend	0.01		0.01		0.03	

Supplementary Table 4. Results of weighted logistic regressions between vitamin B_6 dietary consumption (quintiles) and risk of glaucoma. Model 1 adjusted for age and sex. Model 2 further adjusted for ethnicity, marital status, educational level, household income, total energy consumption, BMI (body mass index), alcohol consumption, waist circumference, diabetes, hypertension, cardiovascular disease, serum total cholesterol, C-reactive protein, dietary consumption of vitamin B_1, B_2, B_3, B_9 and B_{12}. OR (95% CI), odds ratio (95% confidence intervals).

**Vitamin B_6 dietary Consumption, mg/day**	**Glaucoma**					
	Crude model		Model 1		Model 2	
	OR (95% CI)	P	OR (95% CI)	P	OR (95% CI)	P
Q1(≤1.12)	ref		ref		ref	
Q2(1.12-1.50)	1.06(0.54,2.09)	0.86	0.98(0.53,1.81)	0.94	0.83(0.36,1.94)	0.62
Q3(1.50-1.92)	0.74(0.37,1.51)	0.40	0.74(0.34,1.60)	0.42	0.66(0.23,1.91)	0.38
Q4(1.92-2.54)	0.59(0.31,1.13)	0.11	0.51(0.27,0.99)	0.05	0.37(0.17,0.81)	0.02
Q5(>2.54)	0.53(0.30,0.94)	0.03	0.47(0.25,0.88)	0.02	0.29(0.09,0.92)	0.04
P for trend	0.005		0.005		0.01	

Supplementary Table 5. The number of participants with missing values in the confounding variables. BMI, body mass index.

Confounding Variables	Number of Participants	Proportion of Study Population
Marriage	2	<0.001
Household income	116	0.03
BMI	22	0.006
Diabetes	4	0.001
Cardiovascular disease	12	0.003
Serum total cholesterol	5	0.001
**Total**	**161**	**0.04**

Supplementary Table 6. Results of weighted logistic regressions between vitamin B_6 dietary consumption and risk of glaucoma after excluding missing values for confounding variables (n=3689). Model 1 adjusted for age and sex. Model 2 further adjusted for ethnicity, marital status, educational level, household income, total energy consumption, BMI (body mass index), alcohol consumption, waist circumference, diabetes, hypertension, cardiovascular disease, serum total cholesterol, C-reactive protein, dietary consumption of vitamin B_1, B_2, B_3, B_9 and B_{12}. OR (95% CI), odds ratio (95% confidence intervals).

**Vitamin B_6 dietary Consumption, mg/day**	**Glaucoma**					
	Crude model		Model 1		Model 2	
	OR (95% CI)	P	OR (95% CI)	P	OR (95% CI)	P
Q1(≤1.23)	ref		ref		ref	
Q2(1.23-1.70)	0.53(0.30,0.94)	0.03	0.52(0.28,0.96)	0.04	0.50(0.23,1.11)	0.08
Q3(1.70-2.34)	0.51(0.31,0.84)	0.01	0.48(0.28,0.83)	0.01	0.43(0.21,0.86)	0.02
Q4(>2.34)	0.43(0.24,0.77)	0.01	0.40(0.20,0.78)	0.01	0.25(0.07,0.92)	0.04
P for trend	0.01		0.01		0.02	
